# Case Report: Endovascular approach with kissing stent technique in aortoiliac occlusive disease (Leriche syndrome) patient

**DOI:** 10.12688/f1000research.133373.2

**Published:** 2024-07-04

**Authors:** Iwan Dakota, Taofan Taofan, Suci Indriani, Jonathan Edbert Afandy, Yislam Al Jaidi, Suko Adiarto, Renan Sukmawan

**Affiliations:** 1 Department of Cardiology and Vascular Medicine, Faculty of Medicine University of Indonesia / National Cardiovascular Center Harapan Kita / University of Indonesia Academic Hospital, Jakarta, Indonesia; 2 Assistant of Vascular Division, Department of Cardiology and Vascular Medicine, Faculty of Medicine University of Indonesia / National Cardiovascular Center Harapan Kita / University of Indonesia Academic Hospital, Jakarta, Indonesia; 3 Cardiology Resident, Departement of Cardiology and Vascular Medicine, Faculty of Medicine University of Indonesia / National Cardiovascular Center Harapan Kita / University of Indonesia Academic Hospital, Jakarta, Indonesia

**Keywords:** aortoiliac occlusive disease, Leriche syndrome, TASC D, endovascular therapy, percutaneous transluminal angioplasty, kissing stent

## Abstract

**Background:** Aortoiliac occlusive disease (AIOD) or Leriche syndrome, is a peripheral artery disease, specifically affecting the infrarenal aorta and iliac arteries. Presentation of AIOD patients ranged from asymptomatic to having limb-threatening emergencies. Advances and innovations in endovascular devices have replaced surgical approach for AIOD treatment. Here we reported a 52-year-old man presenting with AIOD managed by endovascular approach using kissing stent technique.

**Case presentation:** A 52-year-old man, with history of chronic coronary artery disease, diabetes mellitus type 2, long-standing hypertension, and who was a heavy smoker, was admitted to our hospital with symptoms of long time with bilateral claudication and became leg rest pain. He had history of aorto-infrarenal occlusion known from previous percutaneous coronary intervention from right and left femoral artery access. Aortoiliac occlusive disease (TASC II Type D Class) diagnosis was made by lower extremity duplex ultrasound and CT angiography. The patient underwent urgent percutaneous transluminal angioplasty with kissing stent technique. The patient was discharged 4 days after the procedure without any significant complaints, received optimal medical treatment, and was educated about smoking cessation.

**Conclusion:** Treatment of AIOD should include both modification of risk factors and efforts to increase perfusion to the lower extremities. An endovascular approach is an excellent alternative and may replace surgical approach in complex aortoiliac obstructive disease. In this case report, an endovascular approach with kissing stent technique showed good results for the patient.

## Introduction

Aortoiliac occlusive disease (AIOD), also known as Leriche syndrome results from a chronic occlusive process of the infrarenal aorta and iliac arteries and is the leading cause of peripheral arterial disease (PAD). Epidemiological studies about PAD including AIOD and infrainguinal artery disease have reported that most patients presented with multistage disease. PAD is rare under the age of 50 years, increasing to about 20% by age 60 years and over 40% by age 85 years. An ankle brachial index (ABI) lower than 0.9 is used to diagnose PAD in clinical practice and epidemiologic studies, to identify both symptomatic and asymptomatic patients. ^1^^,^^2^

Risk factors for AIOD include hypertension, hyperglycemia, hyperlipidemia, nicotine use, age, sex, and family history. AIOD patients generally present with a classic triad of clinical symptoms: (1) claudication of lower extremities, (2) impotence, and (3) weak/absence of femoral pulse. Diagnosis of AIOD is made with CT angiography or conventional angiography. Angiography was used to determine the location of the obstruction, length, collateral circulation, and distal patency. ^1^^,^^3^

Advances and innovations in endovascular devices have replaced standard surgical approach with endovascular approach, including in long and complex lesions. An endovascular approach is currently recommended as the definitive interventional treatment for AIOD. Endovascular treatment performed in the aortoiliac segment has resulted in good technical success, low complication rates compared with standard surgery, and offers great patency. Endovascular approach in Trans-Atlantic Inter-Society Consensus (TASC) C and D aortoiliac lesions should be considered as an alternative to the standard surgical approach. ^4^ Here we report a 52-year-old man presenting with AIOD or Leriche syndrome and managed by an endovascular approach using a kissing stent technique in the National Cardiovascular Center Harapan Kita, Jakarta, Indonesia.

## Case report

A 52-year-old Javanese man was referred to our hospital with a history of chronic coronary artery disease, type 2 diabetes mellitus, long-standing hypertension, and who was a heavy smoker. He had symptoms of worsening bilateral leg pain in the past year, and felt chest pain sometimes, especially when he was walking for long distances. Another clinical symptom that he had was impotence. The patient had a history of aorto-infrarenal occlusion that was known when he underwent percutaneous coronary intervention (PCI) *via* the right radial artery but failed after the approach was moved to both sides of femoral artery access because of the occlusion in bilateral common iliac arteries one year earlier. His previous medication was aspilet 80 mg once daily, clopidogrel 75 mg once daily, rivaroxaban 15 mg once daily, bisoprolol 2.5 mg once daily, isosorbide mononitrate 2.5 mg twice a day, candesartan 16 mg once daily, simvastatin 20 mg once daily and novorapid 3×16 IU *sub cutaneously* before meals.

**Figure 1.**
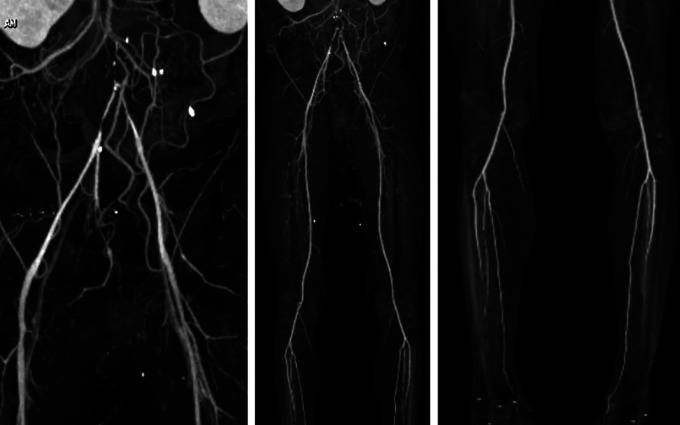
Pre-procedural CT scan angiography.

Physical examination showed blood pressure 160/86 mmHg, HR 68 bpm, RR 18 breaths per minute, temperature 36°C. Normal cardiac, abdominal, and extremity examinations. ECG showed sinus rhythm. Chest radiograph revealed cardiomegaly (65% of cardio thoracic ratio (CTR)) and aorta elongation. Laboratory examination was within normal limits. Lower extremity duplex ultrasound (DUS) suspected significant stenosis of the abdominal aorta at the infra-renal level with high end-diastolic monophasic doppler curve in both external iliac artery and common femoral artery and rounded doppler curve in both popliteal, anterior, and posterior tibial artery, no thrombus in the deep veins in both limbs, and positive arterial flow to distal to both legs. Pletismography examination revealed right ABI was 0.42 and left was 0.35, right toe brachial index (TBI) was 0.45 and left 0.49. Lower extremity CT scan angiography (CTA) showed infrarenal abdominal aortic occlusion from aortic bifurcation, to bilateral common iliac artery and causes suspected thrombus, intermittent atherosclerosis of the abdominal aorta, arterial vasculature of both extremities filled distally, no stenosis or occlusion was seen ( Figure 1).

The patient was diagnosed with chronic limb threatening ischaemia (TASC II type D lesion) causing bilateral aortic infrarenal–iliaca occlusion (Leriche syndrome). The patient was planned to undergo urgent percutaneous transluminal angioplasty (PTA). He got Enoxaparin 60 mg *sub cutaneously* twice per day, some additional anti-hypertension therapy, and other previous drugs were continued.

**Figure 2.**
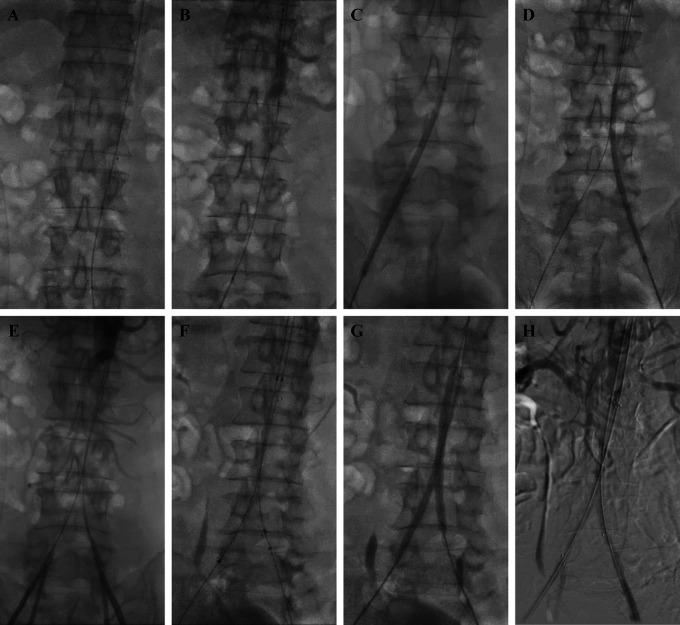
Percutaneus transluminal angioplasty procedure.

The procedure was done by puncturing access via the right brachial artery with a 6F Radial sheath (Terumo, Japan). A JR 3.5/5F diagnostic catheter (Radifocus™ Optitorque™, Terumo, Japan) with support of 0.035 mm exchange wire (Terumo, Japan) was placed in the abdominal aorta above the suprarenal. The aorta blood pressure measurement was 191/74 (120) mmHg. Initial aortography was done and revealed total occlusion in the abdominal aorta from infrarenal the aortoiliac bifurcation until bilateral common iliac arteries ( Figure 2B). Access puncture was then done via both femoral arteries with a 6F Femoral sheath (Terumo, Japan). Blood pressure measurement was done in the femoral artery, the right femoral artery blood pressure was 87/64 (75) mmHg, and the left femoral artery blood pressure was 89/66 (76) mmHg. A 0.035 mm exchange wire (Terumo, Japan) with support of Rubicon 35 Microcatheter (Boston Scientific, MA, US) was used to penetrate the lesion from the right femoral artery and continued from the left femoral artery. Several pre-dilations with 5.0×120×135 mm balloon (Mustang ^®^, Boston Scientific, MA, US) were performed for 10 seconds with a pressure of 4 atm on the right and left iliac arteries ( Figure 2C and D). Then, aortography was performed again and showed positive flow on both right and left femoral arteries ( Figure 2E). Insertion of 12×95×100 mm bifurcated stent graft extension (Seal ^®^, S&G Biotech, Korea) from the right femoral artery access and 12×75×80 mm bifurcated stent graft extension (Seal ^®^, S&G Biotech, Korea) from the left femoral artery access were done ( Figure 2F). Dilatation of the stents was performed with a *kissing stent* technique using a 5.0×120×135 mm balloon (Mustang ^®^, Boston Scientific, MA, US) from the right femoral artery access and a 6.0×100×135 mm balloon (Mustang ^®^, Boston Scientific, MA, US) from the left femoral artery access simultaneously with pressure of 8 − 12 atm for 10 seconds ( Figure 2G). The final result of the procedure was positive flow until the distal part of both common femoral arteries ( Figure 2H) with abdominal aorta blood pressure was 170 / 79 (114) mmHg, right femoral artery blood pressure was 141/68 (98) mmHg, and left femoral artery blood pressure was 137/77 (102) mmHg. The total contrast used was 260 mL iopromide 769 mg/mL, dose area product 418.62 Gy.cm ^2^, and fluoro time was 24.53 minutes.

**Figure 3.**
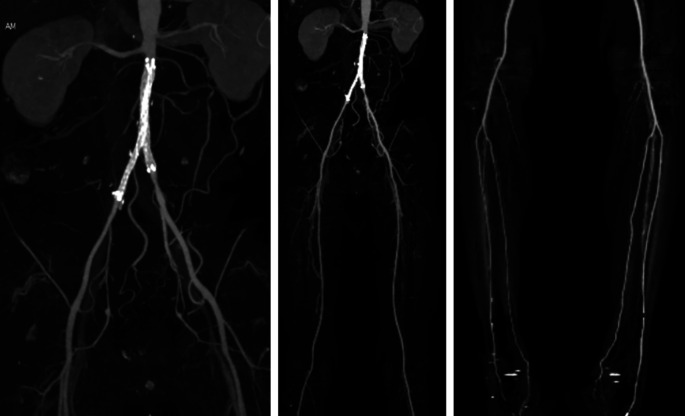
Post-procedural CT scan angiography.

After the procedure, the patient was observed in the intermediate ward. Lower extremity DUS didn’t find any pseudoaneurysm/AV fistula in the right-left femoral region nor deep vein thrombus in both legs, arterial flow was positive to distal of both legs. Plethysmography results were: right ABI 0.8 and left 0.77, right TBI 0.75 and left 0.74. Lower extremity CTA revealed patent kissing stent graft at bilateral common iliac artery, positive flow to bilateral femoral artery, bilateral subpopliteal artery, until bilateral malleolus region especially left anterior tibial artery and right posterior tibial artery. Other contrast flow was improved compared to pre-PTA ( Figure 3). The patient was discharged 4 days after the procedure without any significant complaints, continued his previous medication, and was educated about smoking cessation.

## Discussion

Definitive treatment approaches to AIOD have changed in recent years. Inter-Society Consensus for the Management of Peripheral Arterial Disease suggested that AIOD patients with TASC C and D classification are preferred for surgical treatment, but there has been a shift in the recent guidelines by the European Society of Cardiology and of the European Society for Vascular Surgery suggested that endovascular-first strategy may be considered for AIOD for patient with severe comorbidities and if done by an experienced team. ^5^^,^^6^ Endovascular therapy is a less invasive treatment option and may reduce morbidity. Patients with extensive AIOD could be treated using endovascular techniques with 86% to 100% of the patients' technical success rates. ^7^ Another recent study by Dong *et al*. ^8^ evaluated uninterrupted patency of the treated lesion until 5 years follow-up of AIOD patient treated with endovascular approach was as high as 91.3% and 100% restored blood flow through the original target lesion.

Although several studies have analyzed factors that may affect long-term patency after endovascular treatment of AIOD, including stent placement, lesion morphology, and outflow, there is no consensus currently on the risk factors associated with restenosis after endovascular intervention in patients with AIOD. ^9^ A randomized trial comparing primary *versus* selective stenting for AIOD showed similar long-term patency in both groups, with lower costs in the selective stenting group. ^10^ Nevertheless, most studies for extensive aortoiliac lesions preferred primary stenting. The argument for primary stenting was that stenting without predilatation (direct stenting) reduced the risk of not only vessel rupture but also distal embolism. ^9^ A study by AbuRahma *et al*. ^11^ showed that selective stenting was associated with reduced clinical success in long lesions and that primary stenting should be the option for all TASC II type C and D lesions. In our case, we preferred to do the primary stenting for a better long term clinical succes and patency since our patient was presented with extensive aortoiliac lesions (TASC II type D lesion).

Tegtmeyer was the first to describe bilateral simultaneous balloon angioplasty, known as kissing balloon technique, as a potential endovascular treatment for bilateral proximal common iliac artery stenoses or focal aortic bifurcation. ^12^ However frequent complications occurred, such as dissections and poor angiographic and/or hemodynamic outcomes. Later, the repair of the aortic bifurcation using concurrently placed bilateral stents, known as kissing stents technique, was documented. This made the majority of aortoiliac atherosclerotic lesions amenable to percutaneous therapy. ^13^ Systematic review by Jebbink *et al*. ^14^ revealed that the use of kissing stent technique in AIOD had a 98.7% success rate, 10.8% complication rate, 89.9% clinical improvement achieved in 30 days, and 89.3%, 78.6%, and 69.0% primary patency rate at 12, 24, and 60 months respectively. According to the stent type, Sabri *et al*. ^15^ mentioned that for atherosclerotic aortic bifurcation occlusive disease, covered balloon-expandable kissing stents have greater patency at 2 years compared to bare metal balloon-expandable stents. We used kissing stent technique with covered balloon-expandable stent and showed a very good result. Despite improved blood flow in short-term follow-up by CT scan and duplex ultrasound, long-term follow-up needs to be done for the patient.

Patients with PAD, including those with AIOD, should receive treatment that addresses two different components. First is the risk associated with a particular lesion as mentioned before and second is the aspects related to increased risk of any cardiovascular events. Best pharmacological therapy, as well as nonpharmacological treatment such as smoking cessation, healthy diet, weight loss, and regular physical exercise, should be achieved. ^6^ In our case, the patient was a heavy smoker, but he admitted that he just committed to stop smoking. Unfortunately, he had long-standing hypertension and type 2 DM which also were another important risk factor of atherosclerosis process itself. Good teamwork between care provider, patient, and patient’s family should be targeted in this scenario.

## Conclusion

We had presented a case of a patient with AIOD (Leriche syndrome) TASC II type D which successfully underwent endovascular approach (kissing stent technique) with a good result and blood flow improvement to distal area of lower extremity. Endovascular management of TASC II type C and D lesions approach is an excellent alternative to the surgical approach. Despite that, CV risk modification also has a pivotal role.

## Research ethics and patient consent

Written informed consent has been obtained from the patient for publication of the case report and accompanying images.

## Data Availability

All data underlying the results are available as part of the article and no additional source data are required.
