# Case Report: Endovascular approach with kissing stent technique in aortoiliac occlusive disease (Leriche syndrome) patient

**DOI:** 10.12688/f1000research.133373.3

**Published:** 2024-11-06

**Authors:** Iwan Dakota, Taofan Taofan, Suci Indriani, Jonathan Edbert Afandy, Yislam Al Jaidi, Suko Adiarto, Renan Sukmawan

**Affiliations:** 1 Department of Cardiology and Vascular Medicine, Faculty of Medicine University of Indonesia / National Cardiovascular Center Harapan Kita / University of Indonesia Academic Hospital, Jakarta, Indonesia; 2 Assistant of Vascular Division, Department of Cardiology and Vascular Medicine, Faculty of Medicine University of Indonesia / National Cardiovascular Center Harapan Kita / University of Indonesia Academic Hospital, Jakarta, Indonesia; 3 Cardiology Resident, Departement of Cardiology and Vascular Medicine, Faculty of Medicine University of Indonesia / National Cardiovascular Center Harapan Kita / University of Indonesia Academic Hospital, Jakarta, Indonesia

**Keywords:** aortoiliac occlusive disease, Leriche syndrome, TASC D, endovascular therapy, percutaneous transluminal angioplasty, kissing stent

## Abstract

**Background:**

Aortoiliac occlusive disease (AIOD) or Leriche syndrome, is a form of peripheral arterial disease involving the infrarenal aorta and iliac arteries. The presentation of AIOD ranges from asymptomatic cases to limb-threatening emergencies. Advances and innovations in endovascular devices have replaced traditional surgical interventions for the management of AIOD. Here we report a case of a 52-year-old man presenting with AIOD managed by endovascular approach using kissing stent technique.

**Case presentation:**

A 52-year-old man, with history of chronic coronary artery disease, diabetes mellitus type 2, long-standing hypertension, and a significant history of smoking, was admitted to our hospital with symptoms of long-standing bilateral claudication which recently progressed to rest pain. A history of AIOD was previously established. AIOD (TASC II Type D) diagnosis was made by lower extremity duplex ultrasound and CT angiography. The patient underwent urgent percutaneous transluminal angioplasty with kissing stent technique. The patient was discharged 4 days after the procedure without any significant complaints, received best medical therapy.

**Conclusion:**

Endovascular interventions present excellent alternatives to surgical techniques in the treatment of complex AIOD. Herein we presented an endovascular treatment of AIOD utilizing the kissing stent technique which showed satisfactory outcomes.

## Introduction

Aortoiliac occlusive disease (AIOD), also known as Leriche syndrome results from a chronic occlusive process of the infrarenal aorta and iliac arteries and is one of the cause of peripheral arterial disease (PAD). Epidemiological studies about PAD including AIOD and infrainguinal artery disease have reported that most patients presented with multistage disease. PAD is rare under the age of 50 years, increasing to about 20% by age 60 years and over 40% by age 85 years. An ankle brachial index (ABI) lower than 0.9 is used to diagnose PAD in clinical practice and epidemiologic studies, to identify both symptomatic and asymptomatic patients. ^1^^,^^2^

Risk factors for AIOD include hypertension, hyperglycemia, hyperlipidemia, nicotine use, age, male gender, and family history. AIOD patients generally present with a classic triad of clinical symptoms: (1) claudication of lower extremities, (2) impotence, and (3) weak/absence of femoral pulse. Diagnosis of AIOD is made with CT angiography or conventional angiography. Angiography was used to determine the location of the obstruction, length, collateral circulation, and distal patency. ^1^^,^^3^

Advances and innovations in endovascular devices offered promising alternatives over standard surgical approach, including in long and complex lesions. Endovascular treatment in the aortoiliac segment has shown high technical success and lower complication rates compared to standard surgery. It also provides excellent patency, making it a valuable option to be considered for Trans-Atlantic Inter-Society Consensus (TASC) C and D lesions, especially in patients who are poor candidates for surgery. ^4^ Here we report a 52-year-old man presenting with AIOD or Leriche syndrome and managed by an endovascular approach using a kissing stent technique in the National Cardiovascular Center Harapan Kita, Jakarta, Indonesia.

## Case report

A 52-year-old Javanese man was referred to our hospital with a history of chronic coronary artery disease, type 2 diabetes mellitus, long-standing hypertension, and who was a heavy smoker. Over the past year, the patient experienced long-standing bilateral claudication which recently progressed to rest pain, accompanied by occasional episodes of chest pain, particularly during prolonged walking. Additionally, the patient reported symptoms of erectile dysfunction. The patient had known AOID diagnosed following a failed percutaneous coronary intervention (PCI) attempt. His previous medication was aspilet 80 mg once daily, clopidogrel 75 mg once daily, rivaroxaban 15 mg once daily, bisoprolol 2.5 mg once daily, isosorbide mononitrate 2.5 mg twice a day, candesartan 16 mg once daily, simvastatin 20 mg once daily and novorapid 3×16 IU *sub cutaneously* before meals.

**Figure 1.**
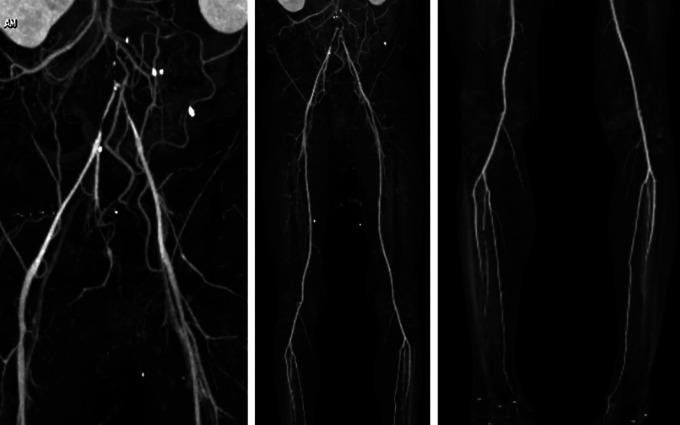
Pre-procedural CT scan angiography.

Physical examination showed blood pressure 160/86 mmHg, HR 68 bpm, RR 18 breaths per minute, temperature 36°C. Normal cardiac, abdominal, and extremity examinations. ECG showed sinus rhythm. Chest radiograph revealed cardiomegaly (65% of cardio thoracic ratio (CTR)) and aorta elongation. Laboratory examination was within normal limits. Lower extremity duplex ultrasound (DUS) suspected significant stenosis of the abdominal aorta at the infra-renal level with high end-diastolic monophasic doppler curve in both external iliac artery and common femoral artery and rounded doppler curve in both popliteal, anterior, and posterior tibial artery, no thrombus in the deep veins in both limbs, and positive arterial flow to distal to both legs. Pletismography examination revealed right ABI was 0.42 and left was 0.35, right toe brachial index (TBI) was 0.45 and left 0.49. Lower extremity CT scan angiography (CTA) showed infrarenal abdominal aortic occlusion from aortic bifurcation, to bilateral common iliac artery and causes suspected thrombus, intermittent atherosclerosis of the abdominal aorta, arterial vasculature of both extremities filled distally, no stenosis or occlusion was seen ( Figure 1).

The patient was diagnosed with chronic limb threatening ischaemia (TASC II type D lesion) causing bilateral aortic infrarenal–iliaca occlusion (Leriche syndrome). The patient was planned to undergo urgent percutaneous transluminal angioplasty (PTA). He got Enoxaparin 60 mg *sub cutaneously* twice per day, some additional anti-hypertension therapy, and other previous drugs were continued.

**Figure 2.**
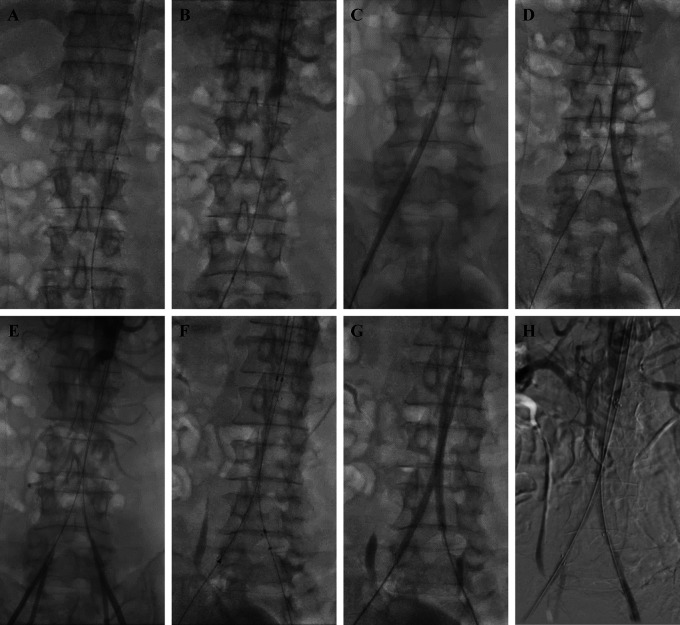
Percutaneus transluminal angioplasty procedure.

The procedure was done by puncturing access via the right brachial artery with a 6F Radial sheath (Terumo, Japan). A JR 3.5/5F diagnostic catheter (Radifocus™ Optitorque™, Terumo, Japan) with support of 0.035 mm exchange wire (Terumo, Japan) was placed in the abdominal aorta above the suprarenal. The aorta blood pressure measurement was 191/74 (120) mmHg. Initial aortography was done and revealed total occlusion in the abdominal aorta from infrarenal the aortoiliac bifurcation until bilateral common iliac arteries ( Figure 2B). Access puncture was then done via both femoral arteries with a 6F Femoral sheath (Terumo, Japan). Blood pressure measurement was done in the femoral artery, the right femoral artery blood pressure was 87/64 (75) mmHg, and the left femoral artery blood pressure was 89/66 (76) mmHg. A 0.035 mm exchange wire (Terumo, Japan) with support of Rubicon 35 Microcatheter (Boston Scientific, MA, US) was used to penetrate the lesion from the right femoral artery and continued from the left femoral artery. Several pre-dilations with 5.0×120×135 mm balloon (Mustang ^®^, Boston Scientific, MA, US) were performed for 10 seconds with a pressure of 4 atm on the right and left iliac arteries ( Figure 2C and D). Then, aortography was performed again and showed positive flow on both right and left femoral arteries ( Figure 2E). Insertion of 12×95×100 mm stent graft (Seal ^®^, S&G Biotech, Korea) from the right femoral artery access and 12×75×80 mm stent graft (Seal ^®^, S&G Biotech, Korea) from the left femoral artery access were done ( Figure 2F). Dilatation of the stents was performed with a *kissing stent* technique using a 5.0×120×135 mm balloon (Mustang ^®^, Boston Scientific, MA, US) from the right femoral artery access and a 6.0×100×135 mm balloon (Mustang ^®^, Boston Scientific, MA, US) from the left femoral artery access simultaneously with pressure of 8 − 12 atm for 10 seconds ( Figure 2G). Following the intervention abdominal aorta blood pressure was 170 / 79 (114) mmHg, right femoral artery blood pressure was 141/68 (98) mmHg, and left femoral artery blood pressure was 137/77 (102) mmHg ( Figure 2H). The total contrast used was 260 mL iopromide 769 mg/mL, dose area product 418.62 Gy.cm ^2^, and fluoro time was 24.53 minutes.

**Figure 3.**
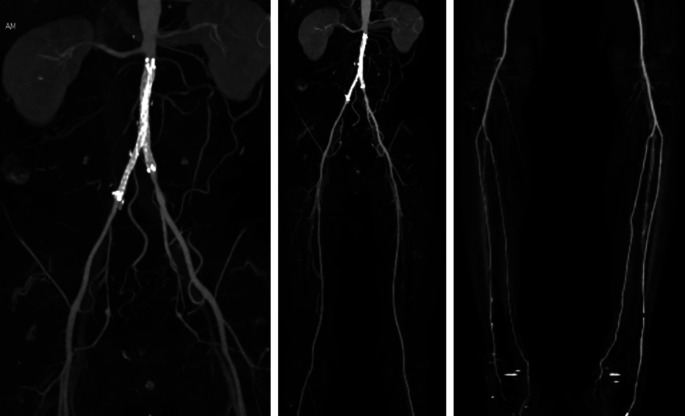
Post-procedural CT scan angiography.

After the procedure, the patient was observed in the intermediate ward. Lower extremity DUS didn’t find any pseudoaneurysm/AV fistula in the right-left femoral region nor deep vein thrombus in both legs, arterial flow was positive to distal of both legs. Plethysmography results were right ABI 0.8 and left 0.77, right TBI 0.75 and left 0.74. Lower extremity CTA revealed patent stent at bilateral common iliac artery, contrast flow was presence to bilateral femoral artery, bilateral infrapopliteal artreies, until bilateral malleolus region especially left anterior tibial artery and right posterior tibial artery. Other contrast flow was improved compared to pre-PTA ( Figure 3). The patient was discharged 4 days after the procedure without any significant complaints, continued his previous medication, and was educated about smoking cessation.

## Discussion

Definitive treatment approaches to AIOD have changed in recent years. Inter-Society Consensus for the Management of Peripheral Arterial Disease suggested that AIOD patients with TASC C and D classification are preferred for surgical treatment, but there has been a shift in the recent guidelines by the European Society of Cardiology and of the European Society for Vascular Surgery suggested that endovascular-first strategy may be considered for AIOD for patient with severe comorbidities and if done by an experienced team. ^5^^,^^6^ Endovascular therapy is a less invasive treatment option and may reduce morbidity. Patients with extensive AIOD could be treated using endovascular techniques with 86% to 100% of the patients' technical success rates. ^7^ Another recent study by Dong *et al*. ^8^ evaluated uninterrupted patency of the treated lesion until 5 years follow-up of AIOD patient treated with endovascular approach was as high as 91.3% and 100% restored blood flow through the original target lesion. Several available reported endovascular techniques for AIOD treatments including covered endovascular reconstruction of the aortic bifurcation (CERAB), unibody bifurcated endografts, and kissing stent technique showed promising patency outcomes. ^9^^–^^11^

Although several studies have analyzed factors that may affect long-term patency after endovascular treatment of AIOD, including stent placement, lesion morphology, and outflow, there is no consensus currently on the risk factors associated with restenosis after endovascular intervention in patients with AIOD. ^12^ A randomized trial comparing primary *versus* selective stenting for AIOD showed similar long-term patency in both groups, with lower costs in the selective stenting group. ^13^ Nevertheless, most studies for extensive aortoiliac lesions preferred primary stenting. The argument for primary stenting was that stenting without predilatation (direct stenting) reduced the risk of not only vessel rupture but also distal embolism. ^12^ A study by AbuRahma *et al*. ^14^ showed that selective stenting was associated with reduced clinical success in long lesions and that primary stenting should be the option for all TASC II type C and D lesions. In our case, we preferred to do the primary stenting for a better long term clinical succes and patency since our patient was presented with extensive aortoiliac lesions (TASC II type D lesion).

Tegtmeyer was the first to describe bilateral simultaneous balloon angioplasty, known as kissing balloon technique, as a potential endovascular treatment for bilateral proximal common iliac artery stenoses or focal aortic bifurcation. ^15^ However frequent complications occurred, such as dissections and poor angiographic and/or hemodynamic outcomes. Later, the repair of the aortic bifurcation using concurrently placed bilateral stents, known as kissing stents technique, was documented. This made the majority of aortoiliac atherosclerotic lesions amenable to percutaneous therapy. ^16^ Systematic review by Jebbink *et al*. ^11^ revealed that the use of kissing stent technique in AIOD had a 98.7% success rate, 10.8% complication rate, 89.9% clinical improvement achieved in 30 days, and 89.3%, 78.6%, and 69.0% primary patency rate at 12, 24, and 60 months respectively. According to the stent type, Sabri *et al*. ^17^ mentioned that for atherosclerotic aortic bifurcation occlusive disease, covered balloon-expandable kissing stents have greater patency at 2 years compared to bare metal balloon-expandable stents. Recent individual participant data meta-analysis by Bontinis, et al. ^18^ also reported improved 48 months patency of covered stent compared with bare metal stent in the treatment of TASC C and D lesions with 92.4% (95% CI 84.7 – 100%) and 80.8% (95% CI 64.5 – 100%) patency rate respectively. We used kissing stent technique with covered balloon-expandable stent and showed a very good result. Despite improved blood flow in short-term follow-up by CT scan and duplex ultrasound, long-term follow-up needs to be done for the patient.

## Conclusion

We presented a case of a patient with AIOD (Leriche syndrome) TASC II type D which successfully underwent endovascular treatment utilizing covered kissing stent technique with a good result and blood flow improvement to distal area of lower extremity. Endovascular approach in TASC II type C and D lesions is an excellent alternative to the traditional surgical technique.

## Research ethics and patient consent

Written informed consent has been obtained from the patient for publication of the case report and accompanying images.

## Data Availability

All data underlying the results are available as part of the article and no additional source data are required.
