# Supplementary material for: Case Report: Endovascular approach with kissing stent technique in aortoiliac occlusive disease (Leriche syndrome) patient
Source: F1000Res. 2024 Nov 6;12:477. Originally published 2023 May 9. [Version 3] doi: 10.12688/f1000research.133373.3 (PMC11550393; doi:10.12688/f1000research.133373.3)
Supplement: Peer review history

# Reviewer report: Reviewer response for version 3

**DOI:** 10.5256/f1000research.174143.r338351

**Published:** 2024-11-09

**Authors:** Vangelis Bontinis

**Affiliations:** 1 Aristotle University of Thessaloniki, Thessaloniki, Greece

The authors have adequately addressed my concerns, and I believe the article is now thoroughly updated and suitable for indexing.

Are enough details provided of any physical examination and diagnostic tests, treatment given and outcomes?

Yes

Is the case presented with sufficient detail to be useful for other practitioners?

Partly

Is sufficient discussion included of the importance of the findings and their relevance to future understanding of disease processes, diagnosis or treatment?

Partly

Is the background of the case’s history and progression described in sufficient detail?

Partly

Reviewer Expertise:

Vascular Surgery

I confirm that I have read this submission and believe that I have an appropriate level of expertise to confirm that it is of an acceptable scientific standard.

# Reviewer report: Reviewer response for version 2

**DOI:** 10.5256/f1000research.168180.r333171

**Published:** 2024-10-25

**Authors:** Vangelis Bontinis

**Affiliations:** 1 Aristotle University of Thessaloniki, Thessaloniki, Greece

General comments - I commend the authors for presenting an intriguing case. However, the manuscript requires careful grammatical revision, as multiple errors are evident throughout the text.

 Abstract

1. “is a peripheral artery disease, specifically affecting the infrarenal aorta and iliac arteries.”

Comment: Rephrase since this English can be improved. A suggestion would be “ .. is a form of peripheral arterial disease involving the….”

1. “Presentation of AIOD patients ranged from asymptomatic to having limb-threatening emergencies “ Rephrase  

Comment: “ The presentation of AIOD ranges from asymptomatic cases to limb-threatening emergencies”

1.  “Advances and innovations in endovascular devices have replaced surgical approach for AIOD treatment. Here we reported a 52-year-old man presenting with AIOD managed by endovascular approach using kissing stent technique.”

Comment: …”have replaced traditional surgical interventions for the management of AIOD. Here we **report a case of a….”**

1. “and who was a heavy smoker”

Comment: “and a significant history of smoking”

1.  “long time with bilateral claudication”

Comment : “long-standing bilateral claudication which recently progressed to rest pain”

1. “He had history of aorto-infrarenal occlusion known from previous percutaneous coronary intervention from right and left femoral artery access.”

Comment: “A history of aortoiliac occlusive disease (TASC II Type D) was previously established”

1.  optimal medical treatment

Comment “best medical therapy (BMT)”

1. “Treatment of AIOD should include both modification of risk factors and efforts to increase perfusion to the lower extremities. An endovascular approach is an excellent alternative and may replace surgical approach in complex aortoiliac obstructive disease. In this case report, an endovascular approach with kissing stent technique showed good results for the patient.”

Comment: You can’t just pop up a comment about risk modification parameters out of nowhere. Then you just performed a case of a kissing stent you can’t claim that just because of this one case endovascular techniques will surpass surgical interventions. These conclusions are merely grounded on your limited persona experience

 A suggested rephrase is “Endovascular interventions present excellent alternatives to surgical techniques in the treatment of complex aortoiliac obstructive disease. Herein we presented, an endovascular treatment of AIOD utilizing the kissing stent technique which showcased satisfactory outcomes.”

 Introduction

  

1.  “is the leading cause of peripheral arterial disease (PAD)”

Comment: About 1/3 of PAD cases present with aortoiliac involvement and very few of them are complete occlusions! The most common distribution of PAD lesions involve femoropopliteal lesions!

  

1. “ nicotine use, age, sex, and family history”

Comment: you mean “male gender”

  

1. “ Advances and innovations in endovascular devices have replaced standard surgical approach with endovascular approach, including in long and complex lesions. An endovascular approach is currently recommended as the definitive interventional treatment for AIOD”

Comment: First avoid repeating the same words within a sentence. Then, no they have not replaced surgical techniques and they are not recommended over surgery. As a matter of fact, this is much more complicated than you suggest with TASC guidelines suggesting endovascular surgery for TASC A and B lesions and surgery for TASC C D lesions (which include occlusions). Additionally, more recent guidelines advocate for endovascular treatment in short lesions or occlusions. So please correct this

 Case report

1. “Javanese man”

Comment: I believe the authors mean "Japanese"

  

1. “ bilateral leg pain in the”

Comment: You mean claudication or rest pain?

  

1. “He had symptoms of worsening bilateral leg pain in the past year, and felt chest pain sometimes, especially when he was walking for long distances. Another clinical symptom that he had was impotence. “

Comment: This English can be improved and a suggested rephrase would be “Over the past year, the patient experienced progressive bilateral leg pain, accompanied by occasional episodes of chest pain, particularly during prolonged walking. Additionally, the patient reported symptoms of erectile dysfunction.”

  

1. “ The patient had a history of aorto-infrarenal occlusion that was known when he underwent percutaneous coronary intervention (PCI)  *via* the right radial artery but failed after the approach was moved to both sides of femoral artery access because of the occlusion in bilateral common iliac arteries one year earlier. “

Comment: This paragraph fails to provide any clarity unfortunately. In my opinion the fact that he had an attempt to PCI and they it failed is of no true value to the manuscript. You can just state that the patient had known AOID diagnosed following a failed PCI attempt.

  

1. “ Insertion of 12×95×100 mm bifurcated stent graft extension (Seal ^®^, S&G Biotech, Korea) from the right femoral artery access and 12×75×80 mm bifurcated stent graft extension (Seal ^®^, S&G Biotech, Korea) from the left femoral artery access were done ( Figure 2F)”

Comment: What I see here are covered stents and not bifurcated grafts which in reality you could not have used so please correct this as this is a major error!

  

1. “ The final result of the procedure was positive flow until the distal part of both common femoral”

Comment: There is no such thing as a positive or negative flow. Omit this while sentence and just state “Following the intervention abdominal aorta blood pressure was 170 / 79 (114) mmHg, right femoral artery blood pressure was 141/68 (98) mmHg, and left femoral artery blood pressure was 137/77 (102) mmHg”

  

1. “ Lower extremity CTA revealed patent kissing stent graft”

Comment: Kissing stents are not grafts they are just kissing stents

  

1.  positive flow to bilateral femoral artery, bilateral subpopliteal artery, until bilateral malleolus region especially left anterior tibial artery and right posterior tibial artery

Comment: Again there is not such a thing as positive flow. There is either presence of flow (monophasic biphasic signal on doppler) or presence or absence of pulses. Please state which one was in your case. There is not an artery called subpopliteal, there are however infrapopliteal artreies (anterio, posterior tibial and peroneal)

 Discussion

  

1. Comment: Endovascular techniques is a general term in your first paragraph you should report on the available endovascular techniques including CERAB and unibody endografts. Also update you reference list accordingly -
  1.  (Bontinis V et al. [2024 (Ref - 1)]) DOI: 10.1016/j.jvs.2023.12.021,
  2. (Manaki V et al. [2024 (Ref - 2)]) DOI: 10.1177/15266028241283721

 

1. Sabri et al.15 mentioned that for atherosclerotic aortic bifurcation occlusive disease, covered balloon-expandable kissing stents have greater patency at 2 years compared to bare metal balloon-expandable stents. We used kissing stent technique with covered balloon-expandable stent and showed a very good result. Despite improved blood flow in short-term follow-up by CT scan and duplex ultrasound, long-term follow-up needs to be done for the patient.

Comment: Update your references list with the latest IPD meta-analysis describing Covered VS Bare for aotoiliac disea with follow-up intervals up to 10 years (Bontinis V et al. [2024 (Ref - 3)]) 10.1016/j.ejvs.2024.06.008

  

1. “ In our case, the patient was a heavy smoker, but he admitted that he just committed to stop smoking. Unfortunately, he had long-standing hypertension and type 2 DM which also were another important risk factor of atherosclerosis process itself. Good teamwork between care provider, patient, and patient’s family should be targeted in this scenario.”

Comment: You are presenting a technical manuscript and this is irrelevant in my opinion

 Conclusion

1. “We had presented a case of a patient with AIOD (Leriche syndrome) TASC II type D which successfully underwent endovascular approach (kissing stent technique) with a good result and blood flow improvement to distal area of lower extremity.

Comment : “We presented”, “covered kissing stent technique”,

  

1. Endovascular management of TASC II type C and D lesions approach is an excellent alternative to the surgical approach The word approach is redundant

Comment : the word approach is out of place here

Are enough details provided of any physical examination and diagnostic tests, treatment given and outcomes?

Yes

Is the case presented with sufficient detail to be useful for other practitioners?

Partly

Is sufficient discussion included of the importance of the findings and their relevance to future understanding of disease processes, diagnosis or treatment?

Partly

Is the background of the case’s history and progression described in sufficient detail?

Partly

Reviewer Expertise:

Vascular Surgery

I confirm that I have read this submission and believe that I have an appropriate level of expertise to confirm that it is of an acceptable scientific standard, however I have significant reservations, as outlined above.

## References

[1] : Covered endovascular reconstruction of the aortic bifurcation: A systematic review aggregated data and individual participant data meta-analysis. *J Vasc Surg* .2024;79(6) : 10.1016/j.jvs.2023.12.021 1525-1535.e9 10.1016/j.jvs.2023.12.021 38104677

[2] : Bifurcated Endografts for the Treatment of Aortoiliac Disease a Systematic Review and Individual Patient Data (IPD) Meta-Analysis. *J Endovasc Ther* .2024; 10.1177/15266028241283721 15266028241283721 10.1177/15266028241283721 39373576

[3] : Editor's Choice - Covered Stents Versus Bare Metal Stents in the Treatment of Aorto-iliac Disease: A Systematic Review and Individual Participant Data Meta-analysis. *Eur J Vasc Endovasc Surg* .2024;68(3) : 10.1016/j.ejvs.2024.06.008 348-358 10.1016/j.ejvs.2024.06.008 38876369

# Response

**Published:** 2024-10-28

**Authors:** Taofan Taofan

**Affiliations:** National Cardiovascular Center Harapan Kita, Indonesia

We would like to thank Dr. Vangelis Bontinis for the constructive insight regarding our manuscript. Here we try to adress and revise our manuscript from Dr. Vangelis Bontinis comments point by point.

**Abstract**

**1. “is a peripheral artery disease, specifically affecting the infrarenal aorta and iliac arteries.” Comment: Rephrase since this English can be improved. A suggestion would be “ .. is a form of peripheral arterial disease involving the….”**

- The sentence has been paraphrased to “Aortoiliac occlusive disease (AIOD) or Leriche syndrome, is a form of peripheral arterial disease involving the infrarenal aorta and iliac arteries.”

**2. “Presentation of AIOD patients ranged from asymptomatic to having limb-threatening emergencies “ Rephrase. Comment: “ The presentation of AIOD ranges from asymptomatic cases to limb-threatening emergencies”**

- The sentence has been paraphrased to “The presentation of AIOD ranges from asymptomatic cases to limb-threatening emergencies.

**3. “Advances and innovations in endovascular devices have replaced surgical approach for AIOD treatment. Here we reported a 52-year-old man presenting with AIOD managed by endovascular approach using kissing stent technique.” Comment: …”have replaced traditional surgical interventions for the management of AIOD. Here we report a case of a….”**

- The sentences have been paraphrased to “Advances and innovations in endovascular devices have replaced traditional surgical interventions for the management of AIOD. Here we report a case of a 52-year-old man presenting with AIOD managed by endovascular approach using kissing stent technique.”

**4. “and who was a heavy smoker” Comment: “and a significant history of smoking”. “long time with bilateral claudication” Comment : “long-standing bilateral claudication which recently progressed to rest pain”**

- The sentence has been paraphrased to “A 52-year-old man, with history of chronic coronary artery disease, diabetes mellitus type 2, long-standing hypertension, and a significant history of smoking, was admitted to our hospital with symptoms of long-standing bilateral claudication which recently progressed to rest pain.”

**5. “He had history of aorto-infrarenal occlusion known from previous percutaneous coronary intervention from right and left femoral artery access.” Comment: “A history of aortoiliac occlusive disease (TASC II Type D) was previously established”**

- The sentences have been paraphrased to “A history of AIOD was previously established. AIOD (TASC II Type D) diagnosis was made by lower extremity duplex ultrasound and CT angiography.”

**6. optimal medical treatment, Comment “best medical therapy (BMT)”**

- The sentence has been paraphrased to “The patient was discharged 4 days after the procedure without any significant complaints, received best medical therapy.”

**7. “Treatment of AIOD should include both modification of risk factors and efforts to increase perfusion to the lower extremities. An endovascular approach is an excellent alternative and may replace surgical approach in complex aortoiliac obstructive disease. In this case report, an endovascular approach with kissing stent technique showed good results for the patient.” Comment: You can’t just pop up a comment about risk modification parameters out of nowhere. Then you just performed a case of a kissing stent you can’t claim that just because of this one case endovascular techniques will surpass surgical interventions. These conclusions are merely grounded on your limited persona experience A suggested rephrase is “Endovascular interventions present excellent alternatives to surgical techniques in the treatment of complex aortoiliac obstructive disease. Herein we presented, an endovascular treatment of AIOD utilizing the kissing stent technique which showcased satisfactory outcomes.”**

- The sentences have been paraphrased to Endovascular interventions present excellent alternatives to surgical techniques in the treatment of complex AIOD. Herein we presented an endovascular treatment of AIOD utilizing the kissing stent technique which showcased satisfactory outcomes.

**Introduction**

**8. “is the leading cause of peripheral arterial disease (PAD)” Comment: About 1/3 of PAD cases present with aortoiliac involvement and very few of them are complete occlusions! The most common distribution of PAD lesions involve femoropopliteal lesions!**

- The sentence has been paraphrased to “Aortoiliac occlusive disease (AIOD), also known as Leriche syndrome results from a chronic occlusive process of the infrarenal aorta and iliac arteries and is one of the cause of peripheral arterial disease (PAD).”

**9. “ nicotine use, age, sex, and family history” Comment: you mean “male gender”**

- The sentence has been paraphrased to “Risk factors for AIOD include hypertension, hyperglycemia, hyperlipidemia, nicotine use, age, male gender, and family history.”

 

**10. “ Advances and innovations in endovascular devices have replaced standard surgical approach with endovascular approach, including in long and complex lesions. An endovascular approach is currently recommended as the definitive interventional treatment for AIOD” Comment: First avoid repeating the same words within a sentence. Then, no they have not replaced surgical techniques and they are not recommended over surgery. As a matter of fact, this is much more complicated than you suggest with TASC guidelines suggesting endovascular surgery for TASC A and B lesions and surgery for TASC C D lesions (which include occlusions). Additionally, more recent guidelines advocate for endovascular treatment in short lesions or occlusions. So please correct this**

- The sentences have been paraphrased to “Advances and innovations in endovascular devices offered promising alternatives over standard surgical approach, including in long and complex lesions. Endovascular treatment in the aortoiliac segment has shown high technical success and lower complication rates compared to standard surgery. It also provides excellent patency, making it a valuable option to be considered for Trans-Atlantic Inter-Society Consensus (TASC) C and D lesions, especially in patients who are poor candidates for surgery.”

 

**Case report**

**11. “Javanese man” Comment: I believe the authors mean "Japanese"**

- Javanese is an ethnic group from Indonesia. It is who we identify as.

**12. “ bilateral leg pain in the” Comment: You mean claudication or rest pain? “He had symptoms of worsening bilateral leg pain in the past year, and felt chest pain sometimes, especially when he was walking for long distances. Another clinical symptom that he had was impotence. “ Comment: This English can be improved and a suggested rephrase would be “Over the past year, the patient experienced progressive bilateral leg pain, accompanied by occasional episodes of chest pain, particularly during prolonged walking. Additionally, the patient reported symptoms of erectile dysfunction.”**

- The sentences have been paraphrased to “Over the past year, the patient experienced long-standing bilateral claudication which recently progressed to rest pain, accompanied by occasional episodes of chest pain, particularly during prolonged walking. Additionally, the patient reported symptoms of erectile dysfunction.”

 

**13. “ The patient had a history of aorto-infrarenal occlusion that was known when he underwent percutaneous coronary intervention (PCI) via the right radial artery but failed after the approach was moved to both sides of femoral artery access because of the occlusion in bilateral common iliac arteries one year earlier. “ Comment: This paragraph fails to provide any clarity unfortunately. In my opinion the fact that he had an attempt to PCI and they it failed is of no true value to the manuscript. You can just state that the patient had known AOID diagnosed following a failed PCI attempt.**

- The sentence has been paraphrased to “The patient had known AOID diagnosed following a failed percutaneous coronary intervention (PCI) attempt.”

**14. “ Insertion of 12×95×100 mm bifurcated stent graft extension (Seal®, S&G Biotech, Korea) from the right femoral artery access and 12×75×80 mm bifurcated stent graft extension (Seal®, S&G Biotech, Korea) from the left femoral artery access were done (Figure 2F)” Comment: What I see here are covered stents and not bifurcated grafts which in reality you could not have used so please correct this as this is a major error!**

- It has been corrected to “Insertion of 12×95×100 mm stent graft (Seal ®, S&G Biotech, Korea) from the right femoral artery access and 12×75×80 mm stent graft (Seal ®, S&G Biotech, Korea) from the left femoral artery access were done.”

**15, “ The final result of the procedure was positive flow until the distal part of both common femoral” Comment: There is no such thing as a positive or negative flow. Omit this while sentence and just state “Following the intervention abdominal aorta blood pressure was 170 / 79 (114) mmHg, right femoral artery blood pressure was 141/68 (98) mmHg, and left femoral artery blood pressure was 137/77 (102) mmHg”**

- The sentence has been paraphrased to “Following the intervention abdominal aorta blood pressure was 170 / 79 (114) mmHg, right femoral artery blood pressure was 141/68 (98) mmHg, and left femoral artery blood pressure was 137/77 (102) mmHg.”

**16. “ Lower extremity CTA revealed patent kissing stent graft”. Comment: Kissing stents are not grafts they are just kissing stents.  Positive flow to bilateral femoral artery, bilateral subpopliteal artery, until bilateral malleolus region especially left anterior tibial artery and right posterior tibial artery Comment: Again there is not such a thing as positive flow. There is either presence of flow (monophasic biphasic signal on doppler) or presence or absence of pulses. Please state which one was in your case. There is not an artery called subpopliteal, there are however infrapopliteal artreies (anterio, posterior tibial and peroneal)**

- The sentences have been paraphrased to “Lower extremity CTA revealed patent kissing stent graft at bilateral common iliac artery, positive contrast flow was presence to bilateral femoral artery, bilateral infrapopliteal artreies subpopliteal artery, until bilateral malleolus region especially left anterior tibial artery and right posterior tibial artery.”

**Discussion**

**17. Comment: Endovascular techniques is a general term in your first paragraph you should report on the available endovascular techniques including CERAB and unibody endografts. Also update you reference list accordingly - 1.  (Bontinis V et al. [2024 (Ref - 1)]) DOI: 10.1016/j.jvs.2023.12.021, 2. (Manaki V et al. [2024 (Ref - 2)]) DOI: 10.1177/15266028241283721**

- Available endovascular techniques have been mentioned in the paragraph: “Several available reported endovascular techniques for AIOD treatments including covered endovascular reconstruction of the aortic bifurcation (CERAB), unibody bifurcated endografts, and kissing stent technique showed promising patency outcomes.”

 

**18. Sabri et al.15 mentioned that for atherosclerotic aortic bifurcation occlusive disease, covered balloon-expandable kissing stents have greater patency at 2 years compared to bare metal balloon-expandable stents. We used kissing stent technique with covered balloon-expandable stent and showed a very good result. Despite improved blood flow in short-term follow-up by CT scan and duplex ultrasound, long-term follow-up needs to be done for the patient. Comment: Update your references list with the latest IPD meta-analysis describing Covered VS Bare for aotoiliac disea with follow-up intervals up to 10 years (Bontinis V et al. [2024 (Ref - 3)]) 10.1016/j.ejvs.2024.06.008**

- Reference has been updated mentioning IPD meta-analysis by Bontinis V, et al. “Recent individual participant data meta-analysis by Bontinis, et al. also reported improved 48 months patency of covered stent compared with bare metal stent in the treatment of TASC C and D lesions with 92.4% (95% CI 84.7 – 100%) and 80.8% (95% CI 64.5 – 100%) patency rate respectively.”

 

**19. “ In our case, the patient was a heavy smoker, but he admitted that he just committed to stop smoking. Unfortunately, he had long-standing hypertension and type 2 DM which also were another important risk factor of atherosclerosis process itself. Good teamwork between care provider, patient, and patient’s family should be targeted in this scenario.” Comment: You are presenting a technical manuscript and this is irrelevant in my opinion**

- The paragraph has been removed from the manuscript

**Conclusion**

**20. “We had presented a case of a patient with AIOD (Leriche syndrome) TASC II type D which successfully underwent endovascular approach (kissing stent technique) with a good result and blood flow improvement to distal area of lower extremity. Comment : “We presented”, “covered kissing stent technique”, Endovascular management of TASC II type C and D lesions approach is an excellent alternative to the surgical approach The word approach is redundant Comment : the word approach is out of place here**

- The sentences have been paraphrased to “We presented a case of a patient with AIOD (Leriche syndrome) TASC II type D which successfully underwent endovascular treatment utilizing( covered kissing stent technique) with a good result and blood flow improvement to distal area of lower extremity. Endovascular approach of in TASC II type C and D lesions is an excellent alternative to the traditional surgical technique.”

Once again, I really appreciate the time and effort that you have invested in reviewing my manuscript. Your insights were helpful, and I am truly grateful for your support and expertise. Please let us know if further revision is needed. Thank you for your contribution to our manuscript.

 Best regards,

 Taofan, MD

 Departement of Cardiology and Vascular Medicine Faculty of Medicine Universitas Indonesia

# Reviewer report: Reviewer response for version 2

**DOI:** 10.5256/f1000research.168180.r299453

**Published:** 2024-07-15

**Authors:** Yopie Afriandi Habibie

**Affiliations:** 1 Department of Surgery, Faculty of Medicine Universitas Syiah Kuala (Ringgold ID: 175503), Banda Aceh, Aceh, Indonesia

Dear Dr. Taofan and Team

 I appreciate your dedication to enhancing this manuscript to advance knowledge.

 Congratulations to Dr. Taofan and the team.

Are enough details provided of any physical examination and diagnostic tests, treatment given and outcomes?

Partly

Is the case presented with sufficient detail to be useful for other practitioners?

Yes

Is sufficient discussion included of the importance of the findings and their relevance to future understanding of disease processes, diagnosis or treatment?

Partly

Is the background of the case’s history and progression described in sufficient detail?

Yes

Reviewer Expertise:

Cardiovascular Medicine, Vascular Surgery, Endovascular, Thoracic Surgery, Cardiac Surgery, Acute Care Surgery, Trauma

I confirm that I have read this submission and believe that I have an appropriate level of expertise to confirm that it is of an acceptable scientific standard.

# Response

**Published:** 2024-09-03

**Authors:** Taofan Taofan

**Affiliations:** National Cardiovascular Center Harapan Kita, Indonesia

Dear Yopie Afriandi Habibie

 I hope this message finds you well.

 I wanted to extend my sincere gratitude for your thoughtful and thorough review of my article, “Case Report: Endovascular approach with kissing stent technique in aortoiliac occlusive disease (Leriche syndrome) patient” which has been approved for publication in F1000research. Your valuable feedback and constructive comments significantly contributed to the enhancement of my work.

 I greatly appreciate the time and effort you invested in reviewing my manuscript. Your insights were incredibly helpful, and I am truly grateful for your support and expertise.

 Thank you once again for your contribution to the successful publication of my article. I look forward to any future opportunities for collaboration.

 Best regards,

 Taofan, MD

 Departement of Cardiology and Vascular Medicine Faculty of Medicine Universitas Indonesia

# Reviewer report: Reviewer response for version 1

**DOI:** 10.5256/f1000research.146357.r252298

**Published:** 2024-05-25

**Authors:** Yopie Afriandi Habibie

**Affiliations:** 1 Department of Surgery, Faculty of Medicine Universitas Syiah Kuala (Ringgold ID: 175503), Banda Aceh, Aceh, Indonesia

**Case Report:**

 1. Please describe the duration of the symptoms and the specific symptoms experienced by the patient during the course related to the AIOD.

 2. Can you provide more specific details about the vascular examination related to AIOD?

 3. In the third paragraph, please correct the diagnosis to Chronic Limb Threatening Ischemia (TASC II Type D lesion).

 4. In the fifth paragraph, please explain how you performed initial orthography in this patient, despite an AIOD? Did you puncture both sides of the Femoral artery? Please explain step by step the procedure before implanting the kissing stent. Also, which wire did you use to penetrate the lesion? For balloon pre-dilatation, which Fr did you use (Fig 2C and D)?

 5. Regarding Fig 2H, do you have another image after the final result showing the stent's patency?

**Discussion**

 1. Paragraph two last sentence: Please explain your reasoning for choosing primary stenting over selective stenting despite the risks.

Are enough details provided of any physical examination and diagnostic tests, treatment given and outcomes?

Partly

Is the case presented with sufficient detail to be useful for other practitioners?

Yes

Is sufficient discussion included of the importance of the findings and their relevance to future understanding of disease processes, diagnosis or treatment?

Partly

Is the background of the case’s history and progression described in sufficient detail?

Yes

Reviewer Expertise:

Cardiovascular, Vascular & Endovascular Surgery, Vascular Medicine, Thoracic Trauma, Trauma, Stem Cell, Cardiac Surgery, Thoracic Disease,

I confirm that I have read this submission and believe that I have an appropriate level of expertise to confirm that it is of an acceptable scientific standard, however I have significant reservations, as outlined above.

# Response

**Published:** 2024-06-20

**Authors:** Taofan Taofan

**Affiliations:** National Cardiovascular Center Harapan Kita, Indonesia

Thank you for the constructive insight into our article. Here we try to answer and revise some of the questions and notes from the reviewer.

**Case Report:**

**1. Please describe the duration of the symptoms and the specific symptoms experienced by the patient during the course related to the AIOD.**

 The patient experienced the symptomps in the past year and his PCI was done one year earlier.

**2. Can you provide more specific details about the vascular examination related to AIOD?**

 Lower extremity duplex ultrasound (DUS) suspected significant stenosis of the abdominal aorta at the infra-renal level with high end-diastolic monophasic doppler curve in both external iliac artery and common femoral artery, rounded doppler curve in both popliteal, anterior, and posterior tibial artery, no thrombus in the deep veins in both limbs, and positive arterial flow to distal to both legs.

 Pletismography examination revealed Rright ABI was 0.42 and left was 0.35, right toe brachial index (TBI) was 0.45 and left 0.49.

**3. In the third paragraph, please correct the diagnosis to Chronic Limb Threatening Ischemia (TASC II Type D lesion).**

 The diagnosis has been revised into chronic limb threatening ischaemia (TASC II type D lesion) causing bilateral aortic infrarenal–iliaca occlusion (Leriche syndrome).

**4. In the fifth paragraph, please explain how you performed initial orthography in this patient, despite an AIOD? Did you puncture both sides of the Femoral artery? Please explain step by step the procedure before implanting the kissing stent. Also, which wire did you use to penetrate the lesion? For balloon pre-dilatation, which Fr did you use (Fig 2C and D)?**

 A more detailed step by step procedure with the device used has been described in the paragraph.

**5. Regarding Fig 2H, do you have another image after the final result showing the stent's patency?**

 We have changed the Fig 2H, hope it can show clearer image of the final result.

**Discussion**

**1. Paragraph two last sentence: Please explain your reasoning for choosing primary stenting over selective stenting despite the risks.**

 In our case, we preferred to do the primary stenting for a better long term clinical succes and patency since our patient was presented with extensive aortoiliac lesions (TASC II type D lesion).

 Once again thanks to Dr. Yopie Afriandi Habibie for the time and effort in reviewing our article, please let us know if further revision is needed.
